# Biomarkers as Biomedical Bioindicators: Approaches and Techniques for the Detection, Analysis, and Validation of Novel Biomarkers of Diseases

**DOI:** 10.3390/pharmaceutics15061630

**Published:** 2023-05-31

**Authors:** Anas Ahmad, Mohammad Imran, Haseeb Ahsan

**Affiliations:** 1 Julia McFarlane Diabetes Research Centre (JMDRC), Department of Microbiology, Immunology and Infectious Diseases, Snyder Institute for Chronic Diseases, Hotchkiss Brain Institute, Cumming School of Medicine, Foothills Medical Centre, University of Calgary, Calgary, AB T2N 4N1, Canada; 2 Therapeutics Research Group, Frazer Institute, Faculty of Medicine, University of Queensland, Brisbane 4102, Australia; 3 Department of Biochemistry, Faculty of Dentistry, Jamia Millia Islamia, New Delhi 110025, India

**Keywords:** biomarkers, biomolecules, omics, proteins, nucleic acids, nanotechnology

## Abstract

A biomarker is any measurable biological moiety that can be assessed and measured as a potential index of either normal or abnormal pathophysiology or pharmacological responses to some treatment regimen. Every tissue in the body has a distinct biomolecular make-up, which is known as its biomarkers, which possess particular features, viz., the levels or activities (the ability of a gene or protein to carry out a particular body function) of a gene, protein, or other biomolecules. A biomarker refers to some feature that can be objectively quantified by various biochemical samples and evaluates the exposure of an organism to normal or pathological procedures or their response to some drug interventions. An in-depth and comprehensive realization of the significance of these biomarkers becomes quite important for the efficient diagnosis of diseases and for providing the appropriate directions in case of multiple drug choices being presently available, which can benefit any patient. Presently, advancements in omics technologies have opened up new possibilities to obtain novel biomarkers of different types, employing genomic strategies, epigenetics, metabolomics, transcriptomics, lipid-based analysis, protein studies, etc. Particular biomarkers for specific diseases, their prognostic capabilities, and responses to therapeutic paradigms have been applied for screening of various normal healthy, as well as diseased, tissue or serum samples, and act as appreciable tools in pharmacology and therapeutics, etc. In this review, we have summarized various biomarker types, their classification, and monitoring and detection methods and strategies. Various analytical techniques and approaches of biomarkers have also been described along with various clinically applicable biomarker sensing techniques which have been developed in the recent past. A section has also been dedicated to the latest trends in the formulation and designing of nanotechnology-based biomarker sensing and detection developments in this field.

## 1. Introduction

“Biomarkers” or “biological markers” are among some of the characteristic features which can be objectively quantified and assessed as a potential indication of any normal or abnormal pathophysiological process or pharmacological response to some therapeutic regimen. Biomarkers are defined as “the substances, structures, or processes which can be quantified in the body or its products and influence or predict the incidences of outcomes or diseases” [1]. The National Institutes of Health (NIH) defines a biomarker as “a characteristic that is objectively measured and evaluated as an indicator of normal biological processes, pathogenic processes, or pharmacologic responses to a therapeutic intervention” [2]. As per the World Health Organization (WHO), the definition of a biomarker covers any evaluation suggesting interactions between any biological system with some potentially hazardous agent, which can be of a chemical, physical, or biological nature. The assessed responses can be of a functional, pathophysiological, and biochemical nature at the molecular, cellular, or histological levels. Examples of some significant clinical biological markers are blood pressure, pulse rate, clinical biochemical indicators, and some other critical more complex laboratory tests of body fluids and several other systems [3]. Biomarkers have always been used in clinical medicines and biomarker-based analyses have now entered new areas and can help in earlier disease diagnoses and efficient therapy of several disorders. These are categorized into five classes depending upon their applicability in various diseases states: (i) antecedent biomarker types, which aid in the identification of risks of disease development; (ii) screening biomarker types, which aid in screening sub-clinical disorders; (iii) diagnostic biomarker types, which help in the recognition of any evident disorder; (iv) staging biomarker types, which aid in categorization of the severity of diseases; and (v) prognostic biomarker types, which give a prediction of the prognosis or course of a disease, such as its recurrence, response to therapeutic paradigms, and monitoring of the effectiveness of therapies. All biomarkers indicate a spectrum of healthy or diseased conditions, which include the levels or types of exposures to various environmental factors, genomic susceptibilities, genetic response to various environmental vulnerabilities, indications of sub-clinical or clinical diseased states, or indications of responses to the therapeutic regimen. Hence, biomarkers are employed for preventing, diagnosing, and therapeutic as well as prognostic purposes, for public health and clinic-based health practices [4]. A biomarker may be present in various forms, which include antibody proteins, microbial indicators, RNAs, DNAs, lipid-based agents, metabolic compounds, and proteinaceous moieties. Changes in their levels, structural aspects, functional behavior, or pharmacological actions are correlated with initiation, progression, and regressive aspects of particular diseases and how patients’ bodies respond to these. Any collective aspect of a dependable and consistent biomarker specific to some diseased state is generally called a biomarker or its molecular signature. The in-depth knowledge and assessment of the importance of these biomarker signatures is significant in the determination of the occurrence, localization, and likeliness of a diseased state. Thus, a biomarker serves as an appreciable and vital tool in detecting, assessing, diagnosing, prognostic, and monitoring various disorders [5,6,7,8,9,10].

Biomarkers were specified by Hulka and co-workers (1990) as “cellular, molecular as well as biochemical alterations which can be assessed and quantified in biological media, viz., human cells, tissues, or fluids” [11]. Today, this definition has been widened for the inclusion of other biochemical features, which are objectively quantified and assessed as the indicators of healthy biochemical procedures, pathological proceedings, or pharmacological outcomes of drug therapy. Today, biomarkers also include tools or techniques which help to understand the prediction, causes, diagnostic aspects, progress ability, regressive features, or responses to treatments of various disorders. Many types of biomarker are employed by physicians, epidemiology experts, and researchers for studying a human’s diseased states. The applicability of biomarkers for diagnosing and managing cardiovascular diseases, immunological diseases, genetic abnormalities, various tumors, and microbial infections, is well-known in today’s times [12]. The first acknowledged biological indicator was the Bence–Jones protein, detected as early as 1847 by precipitating a proteinaceous moiety in an acidic and churned urinary sample. This proteinaceous moiety is made by cancerous plasma-based cellular compartments and is still employed in multiple myeloma detection. Biomarkers are also biological moieties expressed by cancerous cells or normal body tissues, either because of some malignant processes or due to the effects of these cancers. They are indicators that are quantified in higher concentrations in various biological fluids, viz., urine, serum, plasma, whole blood, or other bodily tissue sections of patients with various types of tumors. They comprise a broad range of biomolecules produced in excessive quantities in cancerous cells. These biological markers are conventional endogenous moieties that are produced at greater rates in cancerous tissues or become the product of new genes that are switched off and generally remain silent in normal tissues. A biomarker may be produced by cancerous tissues or by patients’ bodies in response to these cancers. These are present as intracellular products in tissues and can be released into the blood circulation or can show their appearance in serum [13,14].

Biomarkers offer a vital and considerably significant part in enhancing the overall drug developmental processes, in the context of biomedical, therapeutic, and theranostic research enterprises. Implications of biomarkers have become quite general in basic research or clinical practice and their significance as potential and preliminary endpoints in clinical settings has now been well accepted in almost all areas of research [2,3,15]. A joint collaborative report on chemicals safety, called the International Program on Chemical Safety, by the World Health Organization (WHO) in co-ordination with the UN and the International Labor Organization, defines a biomarker as a “substance, structure, or process which is quantified in an organism’s bodies and their formulations and influences or predicts the extent and the incidences of outcomes or diseases” (WHO International Program on Chemical Safety) [16]. Biomarkers also include other aspects, viz., pulse rates and blood pressure readings, from simple chemistry to much more complex laboratory-based tests in biological fluids and other body tissues [17,18]. Biomarkers, when employed as consequences in clinical practice, are acknowledged as surrogate endpoint indicators, which means they can act as surrogate markers or their substituents for clinically significant outcomes. However, these biomarkers do not represent surrogate endpoints, nor are they all intended to be the same. There have been many advantages of using these biomolecules as surrogate endpoints in trials. To recognize these biomolecules as the surrogate outcomes needs the determination of them being relevant and valid [19,20].

## 2. Classification of Biomarkers

The term biomarkers in medicine generally suggests some proteins analyzed in the blood circulation whose amount, in terms of its concentration, demonstrates any normal or pathophysiological responses of the organism, and some pharmacological responses to an applied therapeutical regimen. From a broader viewpoint, the term biomarker is an indication that gives an index about the intensity of some disorder or any other pathological condition inside an organism [21]. This implies that biomarkers have quite a crucial role in medical practices and research to provide an in-depth understanding of mechanisms and courses of disorders. Since many wider spectra of biomarkers are present currently, which can be employed for various approaches, these could be categorized into: (1) antecedence-based biological indicators, which can detect the risks of specific diseases; (2) biomarkers for disease screening, for determination of some sub-clinical forms of disorders; (3) biomarkers for disease diagnosis, which can reveal any existing disorder; (4) staging biomarkers, which can state the stages and severities of these disorders; and (5) biomarkers for disease prognosis, which can establish the course of the disease, including responses to therapies [22]. Regardless of the character, the clinical importance of biomarkers largely depends upon their sensitivities, specificities, prediction values, and the precision with which these can be quantified, their reliability, reproducible nature, and the possibility of feasible and wider applicability. For biomarkers to achieve success, they should pass the processes of validation, relying upon the levels of their application. It is quite crucial for biomarkers, as per their purposes or their characters, to have some specific features for meeting the rigorous necessities for their sensitivities, accuracies, and precision, for their outcomes to be reproduced in the analyses for which these biomolecules are proposed [23]. Lastly, the evolution of regulatory guidelines in applying biomarkers is highly significant, depending upon proper conduction and well-defined evaluation of biomarkers’ assessments, furnishing the criteria through which research can be rendered into clinical practices and can allow the evidence-based facts and figures for the promotion of clinical applicability of novel biomolecules. Depending on the way information is obtained, the following kinds of biomolecules can be categorized: (1) biochemistry or histology-based parameters for detection in tissues procured from biopsy-based procedures or surgeries; (2) biochemical indicators of cells procured from biological fluids; and (3) anatomical, molecular, or functional features which could be demonstrated by bio-imaging technologies [22].

**Figure 1:**
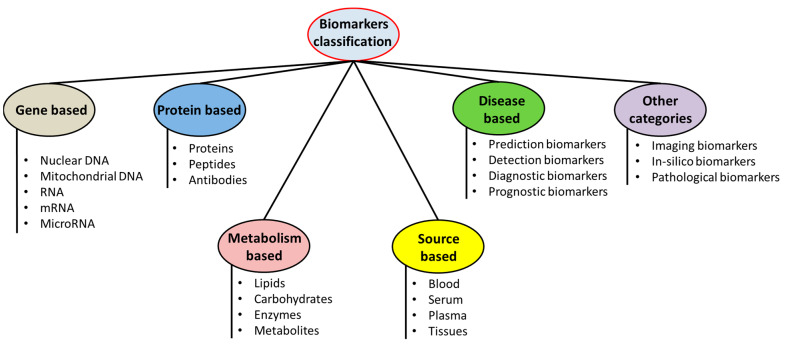
Classification of various biomarkers implicated in health and diseases.

Biomarkers have some characteristic features that can be quantified with objectivity and assessed as the indicators of normal bio-processes, pathological procedures, or pharmacological outcomes of various drug therapies [24]. These are categorized into different types such as (Figure 1):

**(i) Disease or diagnostic biomarkers:** A disease or diagnostic biomarker detects or confirms the presence of a disease or condition or identifies an individual with a disease. Biomarkers which can be correlated with some disorders can be established through biochemical as well as clinical validations. Biomarkers that can be correlated with some disorders when their relations can be established through rigorous biochemical as well as clinical validations. The biomarkers for any disease are not essentially and causally related to all of the disease mechanisms. This correlation with the significant disease phenotypes, and relation to the disease initiation, progress, relapse, or relapses, however, need to be demonstrated. The biomarkers for diseases can play the roles of diagnostic biomarkers, prognostic biomarkers, or as disease classification biomarkers [25,26].

**(ii) Pharmacokinetic or monitoring biomarkers:** When a biomarker can be measured to assess the status of a disease or medical condition for evidence of exposure to a medical or environmental agent or to detect an effect of a medical or biological agent, this is a monitoring biomarker. A biomarker also becomes significant for assessing the distribution of the drug to a particular or targeted location, assessing the residence time of a drug on its target and the extent of modulating or altering the drug targets by its binding or interaction. These are biomarkers that present concentrations of pharmacological drugs in the blood or other body fluids under circulating body fluids and/or at localities of their pharmacological actions, and which are significant for the calculations of their doses, required for the induction of some pharmacological responses [27,28].

**(iii) Pharmacodynamic biomarkers:** When the level of a biomarker changes in response to exposure to a medical or an environmental agent, it can be called a pharmacodynamic or response biomarker. These are biomarkers which demonstrate the functional consequences of various interaction of drugs with their targets (known as pharmacological bioindicators) and can be quantified by an array of techniques (such as enzymology, imaging techniques, omics, etc.). Pharmacodynamic biomarkers are particularly employed for the rationalization of clinical therapeutic efficacies and adverse drug reactions [29,30].

**(iv) Predictive biomarker:** A predictive biomarker indicates that the presence or change in the biomarker predicts an individual’s or population’s exposure to a medical or environmental agent. These biomarkers aid in the potential prediction of patients who may respond to some particular therapeutic regimen or mechanism of actions of drugs or may have some adverse drug reactions [31,32].

**(v) Validated biomarkers:** Biomarkers that can be quantified in analytical testing systems which have well-defined performing characteristic features, and with defined scientific frameworks or a spectrum of evidence that can elucidate the physiological, pharmacological, toxicological, or clinical importance of test outcomes [33,34].

**(vi) Surrogate endpoint:** Biomarkers which can be intended for substitution for some clinical outcomes. The surrogate endpoints are expected to anticipate the clinical advantages (or harms or insufficiency of the benefits or harms) based upon the epidemiological, therapeutical, pathophysiological, and various other scientific evidence [35,36].

**(vii) Therapeutic biomarker:** Biomarkers that can indicate the efficacies of some pharmacological interventions and can evaluate their efficacies and/or safety issues.

**Other important biomarkers:**

(a) **Prognostic biomarkers:** A prognostic biomarker is used to identify the likelihood of a clinical event, disease recurrence, or disease progression in patients with a disease or medical condition.
(b) **Safety**: A safety biomarker is measured before or after an exposure to a medical intervention or environmental agent to indicate the likelihood, presence, or extent of a pathology.

**Susceptibility/risk:** A biomarker that indicates the potential for developing a disease or medical condition in an individual who does not have a clinically apparent disease or medical condition is a susceptibility or risk biomarker [2].

## 3. Biomarkers in Monitoring and Therapy of Diseases

**Figure 2:**
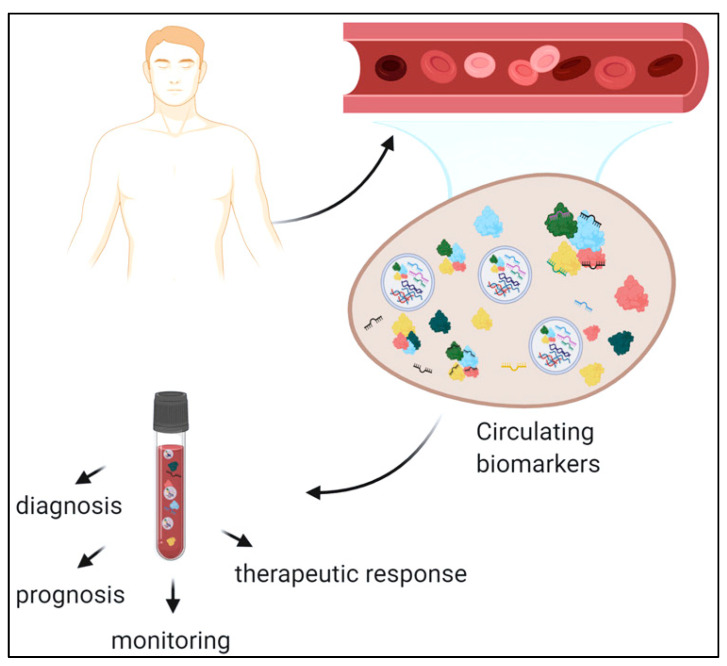
Circulating biomarkers can be employed for disease diagnosis, monitoring, and therapy (adapted from [40]).

A vast variety of biomarkers have been elucidated and measured for the management of almost all diseases and medical conditions or as evidence of exposure to a wide spectrum of medicinal and environmental agents. Proteins, nucleic acids, enzymes, antigens, antibodies, and other biological agents form whole spectra of bioindicators which are assessed for the diagnosis or monitoring of pathophysiological conditions (Figure 2) [37]. For example, the assessment of the therapeutic response in patients of hepatitis C suffering from chronic hepatitis is carried out by measuring the hepatitis C virus RNAs as a monitoring biomarker. Furthermore, in the case of therapy with anti-coagulant drugs, prothrombin time or international normalized ratio is employed as a monitoring marker. Likewise, the level of monoclonal proteins in blood is used as the monitoring parameter in individuals diagnosed with some categories of blood cancer requiring treatment. Similarly, prostate-specific antigen (PSA) has been widely employed as a monitoring biomarker for evaluating therapeutic outcomes in patients of prostate cancers [38].

There is again a variety of cancer antigens, viz., cancer antigen 125 which is exploited for assessment of the disease status or therapeutic management in ovarian cancer patients. In the case of HIV monitoring and therapy, HIV-RNAs have been used as monitoring markers in the case of anti-retroviral therapy. Peptide agents such as B-type natriuretic peptides, as well as N-terminal pro-BNP, have also been exploited as possible biomarkers for follow-up in the clinic-based monitoring of pediatric patients suffering from pulmonary hypertension. In the case of the assessment of patients’ exposure to tobacco smoke or tobacco, the urinary concentration of tobacco-specific nitrosamines as well as 4, (methyl-nitrosamino) 1 (3-pyridyl) 1 butanol have been estimated as potential biomarkers [39].

**Figure 3:**
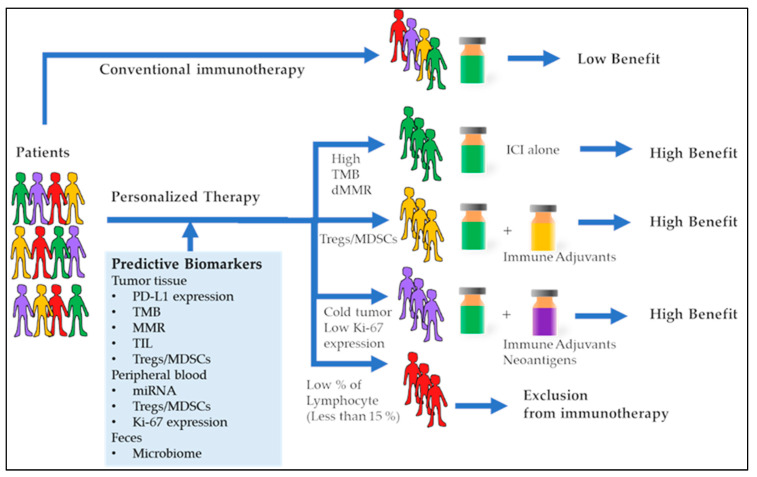
Predictive cancer biomarkers are helpful in providing more effective therapeutic outcomes in personalized cancer therapy as compared to the conventional therapy (adapted with permission from [42]).

Another recent concept is the assessment of biomarkers as predictive biomarkers which can be evaluated and analyzed prior to the start of a therapeutic regimen or during an initial course of drug therapy (Figure 3). Similarly, the assessment of in-treatment biomarkers which can yield an optimized idea of the success or failure of therapy is also equally significant [41]. Immunological biomarkers for diagnosing and monitoring the treatment outcomes of tuberculosis treatment have been under development and can provide robust decision-making capabilities in the context of the treatment course of tuberculosis.

GeneXpert is a recent technology in this regard that detects the DNA in a sample containing both live and dead bacilli in clinical samples. Therefore, Mtb-specific CD_4_^+^ T cells can be correlated with Mtb antigen and may act as potential biomarkers for monitoring anti-TB therapy outcomes [43]. Again, in the case of another microbial infectious disease, viz., leishmaniasis, a need has grown for the availability of pharmacodynamic biomarkers for monitoring and comparison of treatments, especially in the case of visceral leishmaniasis, where a primary outbreak becomes a chronic event and poses difficulty in the disease course prediction. In this case, macrophage-related biomarkers and markers which are directly linked to the parasite burden, and several molecular antigen targets, can sensitively predict the treatment response of leishmaniasis against the anti-leishmania therapy [44].

Another aspect in the context of biomarkers of various diseases is ‘exploratory biomarkers’ that are explored in clinical trials to assess endpoints of several diseases and treatment regimens. These are merely employed for arriving at some conclusive outcomes which can further be analyzed and established as robust endpoints in subsequent clinical-research paradigms. Several kinds of endpoints, viz., a single definite endpoint, multiple-primary outcomes, secondary terminations, and some kind of composite outcomes and consequences of concerned diseases and therapeutic regimens are studied with the aid of these biomarkers [45]. Thus, it can be said that the management of various diseases requires strict monitoring of disease activity in terms of their specific biomarkers to finally assess the due course of treatment. In many cases, this monitoring will provide an optimization of biological as well as non-biological drug agents to be administered and also reduce the uncertainties in the treatment decisions, thereby exerting potential impacts on the economic and clinical outcomes of various therapeutic choices [46].

## 4. Biomarker Analysis

A biomarker is an assessable and quantifiable biomedical parameter that acts as the indicator of a particular pathophysiological condition. A biomarker is a biological molecule whose analysis exhibits some form of a particular disease condition or an effect of a treatment regimen. Examples of these biological indicators include the availability of specified pathophysiological moieties, cellular or tissue-level characteristic features, genetic alterations, or proteinaceous biomolecules. Changes in the levels of some mRNAs and expression levels of some other proteins can also act as biomarkers [47,48,49].

Recent decades of investigations have led to the production of molecular entities which can act as tools for assessing the health quality, epidemiological outcomes, and diagnostic procedures of diseases, and range from tumorigenesis to cardio- and cerebrovascular complications, other neurological disorders, and inflammation-mediated disease conditions. The capability of the efficient treatment of a diseased state frequently depends upon the ability of it to be detected at its earliest stages [50]. Especially in the case of tumors, there has been a dire need for improvement of the early diagnosis paradigms and approaches, because the disorders are frequently detected in later or more advanced stages, which can lead to the postponement of well-times therapy and also leads to a poor prognosis. Enhanced enthusiasm in the assessment of tumor-related risks, and the monitoring of pathophysiological conditions, prediction of their recurring factors, and determination of treatment efficiencies often coincide with the developmental stages in the areas of genome- and proteome-based approaches [51,52,53]. Therefore, biomolecules related to various cancer types have recently been discovered with the application of various technologies, which include DNA-based or tissue-based microarrays, two-dimensional evaluation of gel electrophoresis patterns, mass spectrometric analysis, and protein-based analytics in combination with various advanced-stage bioinformatics-based instruments [54].

**Figure 4:**
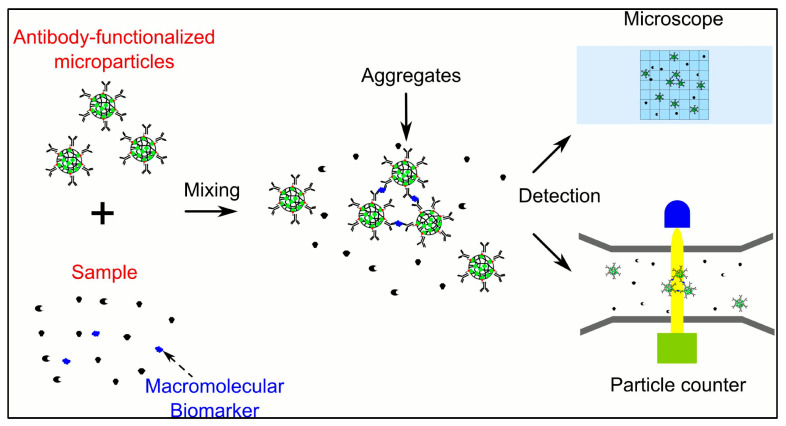
A versatile microparticle-based immuno-aggregation assay for macromolecular biomarker detection and quantification (adapted with permission from [83]).

For the clinic-related implementation or their generalized application, biomarkers should possess high specificity for that particular disorder and should be quantifiable with ease inside the approachable body fluids, including but not limited to serum, sweat, urine, or saliva. A tumor biomarker molecule, for example, can have an association with a body’s responses to tumor progression or metastasis, or it can also have secretions from the malignant cells or tissues themselves and can be feasibly observed from the body’s fluids. Examples of frequently employed tumorigenesis markers include cancer-specific antigens, viz., CA15-3, as exemplified in cases of breast tumors, CA125 which can be detected in ovarian cancer patients, and prostate-specific antigens, etc. [55]. These biomarkers and their quantification testing provide considerable aid in the diagnoses of circumstances for monitoring the pathophysiological processes, stages of the disease development or progression, assessment of the disease prognosis, guidance and monitoring of the therapeutic regimen, and determination of recurrence of the disease. Biomarker evaluation becomes a valuable case for disease diagnosis, monitoring prognostic conditions, and monitoring the stages of disease development, as in the case of tumor progression or metastasis. Once patients become affirmative for some disease-specific biomarker, before the therapy initiation, the efficient clinical application can be manifested only after continuous quantification during the entire clinical therapy of patients [56,57]. Certain biological markers are isolated by utilization of immunohistochemical staining techniques, polymerase chain-reaction-based analysis, ELISA-based immunoassays, and other immuno-aggregation or immunoprecipitation assays, etc. (Figure 4).

The diagnostic efficacy of a biomarker is based upon a variety of components related to its specificity, sensitization capability, and can be positive as well as negative [58]. Sensitivities, in the case of cancer biomarkers, become the probabilities of their observations in tumor-bearing patients. Specificities of cancer biomarkers for their screening values exhibit whether these can be applied to the description of what fraction of normal healthy subjects will appear negative for the test results. Positive predictive values here suggest the probability of the test subject having the disorder under consideration if the tested outcomes are positive. Likewise, negative predictive values (NPV) suggest the probability of the test subject having a disorder if the tested outcomes are negative. Currently, there is a dearth of some ideal biomarkers that are suitable for generalized screening procedures, although some of those can be applied for monitoring persons who possess a stronger familial historical background for a specific kind of cancer. Genetic biomarkers can also aid in the prediction of risks associated with other family members. For those patients possessing particular symptoms, biological markers may be employed for the identification of a specific tumor source [59].

For instance, CA-125 can be analyzed in patients with ovarian cancers, and thus can aid in their differentiation and distinction from several other cancer types. When a patient has tumors, a rise in the levels of some biomarkers can aid in the determination of progression, metastasis, and spreading of the cancer to other organs or tissues. Other biomarkers can be employed for the determination of tumor aggression and can yield useful and in-depth knowledge related to what kind of therapy needs to be administered to patients to obtain the best response [60]. For example, patients with breast cancer positive to Her2/neu factors will have a stronger and positive response to herceptin-inclusive therapies. Biomarkers are also used for monitoring the efficacy of therapies, especially in advanced stages of tumors. However, the quantification of almost all the tumor biomarkers will generally be insufficient for the tumor diagnosis [61]. This is because their level could be enhanced in patients who possess benign circumstances. Furthermore, biomarkers of cancer may not be increased in every patient having the tumors, particularly in the initial developmental stages of this disorder. Various biomarkers of cancers are not specific to a particular tumor type and their levels could well also be increased in more than one tumor type [62,63]. A technique that holds promise in the discovery of various biomarkers is the combination of protein array and SELDI-TOF-MS (surface-enhanced laser-desorption ionization time-of-flight mass spectrometric analysis). This paradigm can distinguish between the presence and absence of disease by analyzing the complex proteins as well as their various mixtures, and expression level variations among various protein molecules. Application of computational methodologies can generate enormous quantities of proteomics spectral data, which can then be superimposed for the detection of alterations in the proteinaceous expressional levels and their linkage with a particular disease’s condition [64,65,66,67].

A biomarker can be used for disease diagnosis if the material where it is assessed can be feasibly procured. It could well be a serum, urine, saliva, or blood sample, or various other biologic materials. The methodology applied for determining the biomarker needs to be accurate and be able to be performed easily. The outcomes and results must be preferably retrieved in short periods and should not indicate any inter-laboratory variations, while the biomarkers should be employed efficiently for disease diagnoses, prognoses, and risk evaluation in various patients [12]. Several of these biomarkers are applied in molecular medicines, for whose evaluation and quantification various methodologies are undertaken. Several expression levels are employed for signifying the approaches for the determination of these biomarkers, viz., genomics, lipidomics, metabolomics, and glycomics, etc. (Table 1). Expression-metabolomics, or sometimes metabolomics, generally implies the analyses of all metabolic products in a biological sample. Lipidomics, however, signifies the analyses of lipidic metabolic products and lipid-based substances by various tools and techniques such as mass spectrometric analysis, chromatographic assessments, nuclear-magnetic-resonance-based assays, etc. [68,69]. Genomic approaches include techniques such as northern blotting, genetic expression analysis, DNA microarray-based assays, and SAGE, etc., while proteomic approaches include SELDI-TOF, LS/MS, 2D-PAGE, antibody-based microarray techniques, and tissue-based microarrays. The combination of bio-medical imaging techniques and other chemical biomarkers is particularly prescribed for initial cancer diagnosis and for further advancements of diagnosis and therapy paradigms [70,71,72]. Several new biomolecules have recently been researched for employment in imaging techniques. They usually offer non-invasive approaches and are characterized both qualitatively and quantitatively and yield multi-dimensional outcomes. In combination with other knowledge, these can be used by clinical researchers in the establishment of disease diagnoses, particularly in developing bio-imaging technologies in cardiographs and cardiac-specific computerized tomographic analysis [73,74]. Nowadays, it has become feasible to diagnose benign as well as malignant tumors by using ultrasonography, computerized tomographic analysis, and MRI evaluations based on discovering morphological changes in patients’ bodies. These technologies permit the study of morphological changes or functional alterations in various pathophysiological pathways. Conventional bio-imaging markers have largely been based upon nuclear imaging techniques, viz., scintillations, photon emission tomographic evaluations, and PET, etc. [75,76]. For biomarkers to be credible, they must possess the capabilities of validation and, as per the nature of their applicability, certain features must be present to meet the genuine necessities of accurate estimations, sensitivities, and precise measurements. Hence, various groups of researchers have recommended the regulatory procedures and have laid down guidelines for the evaluation of these biomolecules for prognosis as compared to diagnosis paradigms [22].

Various technological advancements employed in biomarkers research include contemporary in vitro analysis of biomolecules such as DNA alterations, RNA expressional changes, protein analysis, and metabolic biomolecular quantifications, and in vivo quantification of biomolecular proceedings in both animals and humans through morphometric and functional bio-imaging techniques. The importance of quantification relates to the prediction capabilities of biomolecules in clinically-relevant outcomes, viz., disease stages, outcomes of various therapeutic regimens, and prognostics of diseases [77,78]. The characteristics of clinic-based population samples at the molecular level are among some of the goals of biomarker discoveries and feed drug developmental procedures. The application of biomolecular markers requires not only the study of in vitro procedures but requires technological advancements which are applicable to a greater number of patients. It thus becomes significant that such techniques employed for biomarker discovery are often not similar to those technologies which are employed in clinical settings. A massive scaled parallel analyzing technology needs to be used for screening the individualized biomarker specimen [79,80,81]. Once these biomolecules are analyzed and quantified, other simple and more economical techniques are employed for analyzing the clinical specimen. One of the challenges which becomes evident when many biomolecules are characterized, is that the most economical and fastest analysis tools are often not optimized or cost-effective for the multiple testing of these specimens [82].

As defined by the National Cancer Institute, biomarkers are biological molecules found in body fluids such as blood, etc., and in other body tissues which can offer the signs of some normalized or abnormal pathophysiologic conditions, or a disease. Biomarkers generally become differentiated in a patient suffering from a disease as compared to other persons without the same disorder. These changes can arise because of various factors, which include somatic or germinal mutational changes, transcriptional alterations, and post-translational changes [13,84]. There are tremendous varieties of biomolecules, including proteinaceous moieties (viz., enzymes and receptors), nucleic acids (viz., mRNAs and non-coding RNAs), antibody-based markers, peptide-based molecules, etc. Biomarkers can also refer to some collections of changes, such as genetic expressional changes, protein-based alterations, and changes in metabolic signatures [84,85]. Biomarkers can also be observed in blood (either in whole blood, plasma, or serum) or the body’s secretions and excretory materials (saliva, sputum, urine, or stool) and hence can feasibly be evaluated in a non-invasive manner; or, they can also be a tissue-derived substance and require biopsy-based procedures or specific bio-imaging technologies for their assessment. Genetically significant biomolecules can also be passed on via inheritance, and can be quantified as sequential changes in germline DNA separated either from whole blood, body tissues, or it can also be of a somatic nature, and described as mutational alterations in DNA obtained from cancer cells [84].

## 5. Approaches for Biomarkers Detection and Analysis

**Figure 5:**
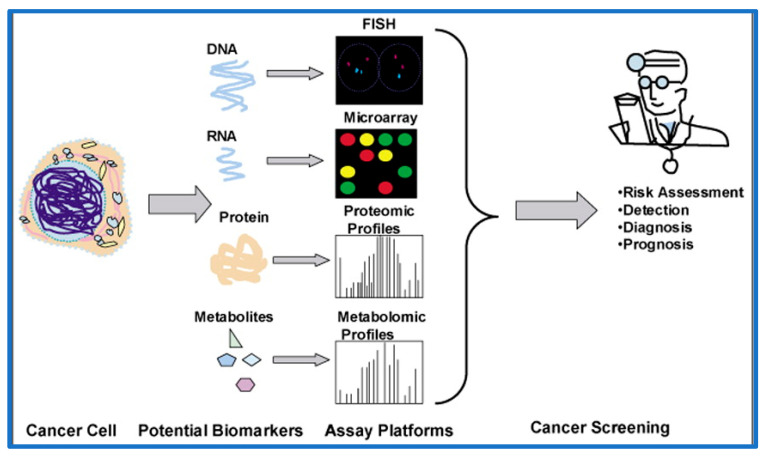
Various approaches for biomarker detection and analysis assays (adapted from [93]).

A wide array of technologies and protocols have been developed for the detection and analysis of biomolecules and biomarkers, and this has enabled the gathering of a massive amount of data for the characterization of these biomolecular markers in a complex mixture of proteins, carbohydrates, lipids, and nucleic acids (Figure 5). A comparative analysis of bio-fluids such as whole blood, plasma or serum, semen, urine, saliva, and sweat, etc., collected from healthy as well as disease-affected individuals, can reveal some of the very highly specific biomarkers on some very sensitive diagnostic platforms. In this regard, proteomics plays an integral and vital part in mass spectrometry (MS)-based diagnostic techniques, where large efforts have been put into the optimization of these proteomics technologies which can have an important impact on the diagnosis of diseases [86].

Proteomics research has further been advanced by the availability of human and other pathogen genome sequences, and this contributed a lot towards the identification and characterization of biomolecular markers in their initial diagnoses and monitoring of tumors as well as other fetal diseases. Still, the critical detection of lower abundance biomolecular markers in complex biological milieu and under emergency conditions needs the development of ultrasensitive, robust, and high-throughput techniques. Restrictions of sensitivity, detection time, dynamic biomarker range, and multi-complexation have led to the development of nanotechnology-based sensing and detection platforms which has further paved the way for the formulation of ‘nanoproteomics’, leading to many classes of nanomaterials such as metallic and polymeric nanoparticles, carbon nanotubes, quantum dots, and nanowires coming into research practices [87,88,89]. Further down the line in proteomics, the detection and analysis of sensitive amino acid-based biomarkers with ultrasensitive techniques such as the Mars organic analyzer (MOA), micro-fabricated capillary electrophoresis instruments, glass separation channels, micro-fabricated pneumatic membranes and pumps, etc., has become possible. Other techniques, such as fluorescence detection optics, can detect amino acids in concentrations as low as 0.1 nM, which corresponds to parts per trillion sensitivity [90]. Furthermore, these ‘omics’ approaches have also contributed to the discovery of novel biomarker biomolecules, which can be otherwise referred to as molecular signatures of diseases, and provide highly significant additional value in clinical practices. These omics-based approaches, which include genomics, transcriptomics, metabolomics, and proteomics are investigated in the context of many pathophysiological disorders and employ advanced tools for investigating alterations in proteins, peptides, nucleic acids, and other metabolic biomolecules [91]. Advances in two-dimensional electrophoresis, antibodies and protein arrays, and mass spectrometry have aided in the search for potential biomarkers and their characteristic “fingerprint” profiling. Furthermore, significant improvements in tandem mass spectrometers, and laser capture microdissection microscopes have greatly paved the way for the detection, identification, characterization, and analysis of many of the vital prognostic and diagnostic biomarkers of various diseases [92]. Various technological advances in the context of biomolecular detection, recognition, and analysis are further discussed in detail in the following section.

### 5.1. Surface Functionalization for Biomolecular Recognition

Recognizing biomolecular entities plays a significant role in designing and surface functionalization of biosensors and many of the nano-structural components have been modified, served as promising candidates, and attracted a significant degree of attraction in the recent past. In this regard, the recognition of specific biomolecular interactions in the diagnosis of disease conditions via biosensor biomolecules and the determination of their concentrations in the biological milieu can indicate disorders such as cancers, inflammatory disorders, and other lifestyle diseases. For analytical specifications, the binding event of the targeted biomarker is converted into a detectable signal, and this surface-modified detecting platform offers highly specific and selective detection of the target analyte [94,95].

**Figure 6:**
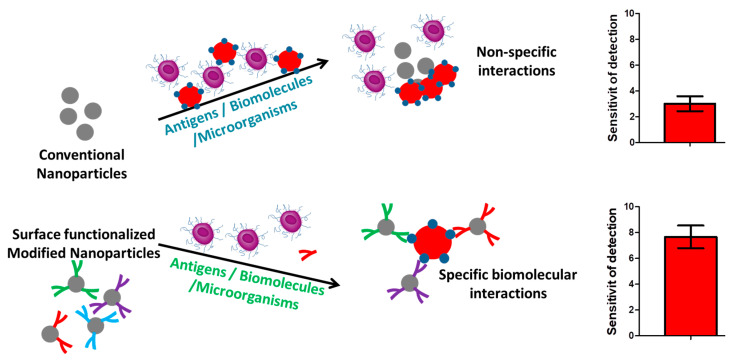
Surface functionalization of sensing nano-biomaterials leads to more specific substrate binding and, further, results in enhanced sensitivity of detection.

Another approach, called biofunctionalization, undertakes the modification of the surface of interest by immobilizing various biomolecules, viz., proteins, peptides, and polysaccharides, as well as certain bioactive drugs, for the purpose of biomolecular recognition (Figure 6). Biofunctionalization is also performed with the aim of quantifying the targets such as proteins in assays, cellular compartments, and tissue homogenates. In this regard, surface modification of nanoparticles for surface-enhanced Raman-scattering-based detection can significantly improve their detection capacities and target specificities. The integration of surface peptides for surface modification or functionalization can appreciably aid in the recognition of integrin motifs, cyclic RGD peptides formed on the surface of integrin-based cellular adhesion platforms [96,97].

Despite having great advantages, the functionalization approaches for biomolecular recognition possess a number of lacunae, which include difficulties in real-world applicability due to lower analytical specificity and sensitivity in their molecular recognition capabilities. The modifying features of associated antibodies with a high number of multistep modification procedures and the adsorption of undesired and non-specific proteins from the biological fluids over these modified antibodies also offer a significant degree of limitations in the context of their usability in clinical settings [98,99,100]. To address these lacunae, Shimada et al., proposed surface-modified nanowires for biomolecular recognition of C-reactive protein (CRP), by formulating self-assembled monolayers enabled by thiolated 2-methacryloxyethyl phosphorylcholine. These self-assembled monolayers significantly suppressed the non-specific biomolecular adsorption on the nano-formulation and led to the highly specified capturing of only targeted proteins in the Ca^2+^ ions’ presence. Furthermore, another reward of this approach is the recognizing capability of the biomarker protein on the nanowires’ surfaces, which was altered through a single-step modification procedure [101].

In a recent approach, Oliverio et al. described the limitations associated with the use of conventional biosensors that are composed of two areas, one main area for sensing and another outer surrounding locality that can be hardly functionalized and often poses the problems of cross-talk. This kind of lacunae can be minimized by designing some highly specific biosensors with higher analytical recognition capabilities depending upon the orientation of the biomolecules under question, which can act as specific analyte receptors with better functional capabilities [102]. Another report, by Sonawane et al., discusses the various chemical approaches for the surface modification and decoration of nanomaterials employed in disease diagnosis procedures and uses various biomolecules including, but not limited to, enzymes, DNA, and proteins for the particular approach. The performance of biosensing platforms largely depends upon the kind of substrate materials and their modifiable chemical properties. The surface functionalization of biosensors for optimizing the binding efficacy of biomolecules, in addition to the maintenance of their biological activity, is a phenomenal achievement and also aids in the key detection precision. Biosensors are susceptible to a number of surface modification techniques such as covalent customizations, electrochemical tailoring with silicon materials, physisorption-dependent modifications, and, covalent customization with glass materials, aldehydes epoxy, and carboxylate modifications, etc., are some of the novel techniques that are highly efficient in enhancing the surface properties of biosensors for the detection of analyte biomolecules [103]. Surface functionalization of gold-based materials with cellulose and cellulose derivatives can also lead to an improvement in their sensing abilities via in situ reduction in gold precursors, its deposition, or via its covalent fixation in the context of its nanoparticles. An alternate strategy is to selectively functionalize gold with biotin molecules for biomolecular recognition that considers carbohydrate binding moieties as well as a Z fragment from the staphylococcal protein. The biomolecular recognition reaction will yield a red color in the case of modified and functionalized gold nanoparticles, whereas no color will be present in case of the conventional unmodified gold nanoparticles. This approach paves the way for the formulation of a simpler and straightforward platform for the direct detection of the analyte molecules by employing the color base signaling protocol [104]. Another approach for surface modification of nanomaterials for biomolecular recognition properties involves carbon nanofibers, such that these are covalently or non-covalently modified either by electrochemical or photochemical methods and respond to the presence of free amine groups. These nanofibers are then capable of binding to various DNA molecules as well as thiol-terminated oligonucleotides. This technique has proved quite useful for the formulation of biologically modifiable carbon-based nanofibers possessing excellent electrochemical properties and can be exploited for the preparation of nanomaterials for electrical sensing of some specific biomolecules in biological solutions [105].

MicroRNAs (miRNAs) have now been recognized as quite appreciable tools as blood biomarkers, and methods are being developed for the quantification of miRNA levels which can monitor their expression levels in ultra-low concentrations in the biological milieu. In this regard, gold-coated magnetic nanoparticles were modified by probe DNAs for the creation of nanosensors with very high specificity for direct measurements of nucleic acids in a patient’s whole blood. These nanosensors were capable of detecting miRNA concentrations as low as 1–10 nM in blood and could also differentiate between different types of miRNAs obtained from various tumor tissues. This ultra-sensitive and straightforward assessment of miRNAs by employing electrochemically reconfigurable modified DNA-based nanosensors offers a promising strategy for cancer detection as well as in theranostics [106].

### 5.2. In Situ Analysis of Molecular Interactions

The in situ analysis of various biomarkers in biological fluid samples can be performed with various techniques, and with the passage of time great advancements have been achieved in the context of in situ detection and the qualitative and quantitative assessment and evaluation of various generalized as well as disease-specific biomarkers. Real-time visualization of the change in biomarker concentration has also been achieved with the aid of various biomolecular interactions. This approach of biomarker detection uses the transduction and modulation of biomolecular affinity on the surface of biomolecular sensors, and this modulation is brought about by the target biomolecule which is precisely bonded to the molecular recognition sites [107]. This paradigm has been designed in such a way that laser lights undergo scattering by specific bonded biomolecules and thus generate stringer signals, while non-specific and non-bonded biomolecules do not bestow those particular effects. In this context, Gatterdam et al. have presented a detection and analytical approach via a chip that utilizes a near-field reaction immersive lithographical concept on a light-sensitive grafted copolymeric layer. They showed that very specific and highly sensitive biomolecular detection takes place when these biomarker molecules are bonded to the recognition sites and form many biological complexes in the sample solution. Their strategy also facilitates label-free detection as well as analysis through non-covalent interactive forces in the complex biological milieu and it also eliminates the necessity of rigorous sample preparation, permits time saving, and offers an economical approach for the development and performance of immunological assays and diagnostic procedures [108].

Another approach elucidates the biomolecular interaction of magainin-2 will modular cellular membrane and employs frequency-generating vibration spectroscopic technique and ATR-FTIR phenomenon. It was demonstrated by the authors that biomolecule magainin-2 becomes oriented in a parallel fashion to that of the lipid bilayer and interacts with the cellular membranes. It is possible to quantitatively estimate the lower biomolecular concentrations as low as that of the 200 nm. In this technique, researchers have provided ample investigative proof that sum frequency generation provides comparatively much better detection capabilities than that of the ATR-FTIR approach and thus can be a much better paradigm for studying the biomolecular interactions at the interfacial regions with much lower surface coverings [109].

Likewise, Kunneke and co-workers formulated a generalized microstructural procedure for the generation of various specific and selective functional lipid-based membranous compartmentalizations based on solidified supports located in close proximity to each other. Their approach enabled highly specific recognition ability for saccharides-based structures located on cellular surfaces, and the distinguishing of normal as well as malignant cell kinds. It also enabled the binding of certain toxic proteinaceous materials such as cholera toxins, diphtheria toxins, toxins from botulinum, tetanus toxins, etc., and other biomolecular markers of inflammation processes [110].

Amperometry-based enzymatic channel-dependent immune-sensing agents have been conceptualized for studying the quantitative and qualitative real-time estimation of biomolecular recognitions and interactions. These sensors use the approach of polyethyleneimine-based carbon material electrodes which can be exploited for the immobilization of glucose oxidase enzymes and certain highly specific antibodies. With these sensors, immunological reactions were carried out, monitoring was followed in situ through electrochemical methods, and the curves for the binding energy were plotted and could be demonstrated on computer screens. This approach can be employed for the estimation of the kinetic energy and the constant reactions between immunoglobulins (IgG) and their highly specific anti-IgG antibodies. In this way mapping of the various sites of biomolecular interactions and their target peptides as well as proteins through their particular antibodies can be carried out [111]. Manolova and co-workers have exploited the interactive forces between ATP and some self-assembling monolayers using an electrochemical method and employed a combinatorial strategy based upon an electron spectroscopic methodology as well as a few computer-based simulations. They provided some evidence for the formulation of a biomolecular double layer in those sensors. They elucidated that stronger chemical interactive forces between metals and other molecules of organic origin can provide a great deal of in-depth knowledge and deeper insights into the qualitative and quantitative detection of biomolecules in the solutions, and this knowledge can be brought about by closely monitoring the tunneling resistance between metallic electrodes which happens after the biomolecules are absorbed [112].

### 5.3. Specific Affinity Determination in Direct Binding Assays

Generating the multi-dimension-based spatial expressional mappings of biological molecules directly from a tissue section can be achieved by a powerful tool known as matrix-assisted laser desorption ionization (MALDI) imaging mass spectrometry (IMS). By this methodology, the different analytical approaches and spatial locations of several of the biomolecules, right away from the sections of tissues obtained from patients, can well be accomplished. For example, in cases of breast cancers, HER2 and/or Neu oncoprotein overexpression has been one of the significant factors correlated with a poor medical prognosis [113].

For establishing the derived functional expression between cancerous and non-cancerous tissues of prostate cancer patients, the higher expression of an individual peptide at an m/z ratio close to 4355 was discovered, and was employed for the accurate substitution of cancerous tissues from the contiguous healthy tissues. Biomarkers which are obtained specifically for a disease or several phases of these diseases can appreciably allow surgeons, pathologists, and physicians to provide their patients with a “personalized” diagnostic and prognostic paradigm [114]. Cardiovascular disease (CVD) is a major known cause of human death in both developing as well as developed nations. Recently, microfluidics and lab-on-chip-based biosensors have been evaluated for detecting cardiac biomarkers. The early assessment of the creatine kinase MB sub-forms (CK-MB) for the detection of acute myocardial infarction (AMI), has been employed in assessing CVD. Detection of inflammation in cardiovascular diseases can be assessed by myeloperoxidase (MPO). Furthermore, detection of acute myocardial infarction (AMI) can be detected by Troponin 1(cTnI) [115].

### 5.4. Therapeutic Antibody Detection in Human Serum

It has been immensely significant to detect various types of biomolecules in human tissue and fluid samples for the detection, diagnosis, or prognosis of various diseases. A number of versatile approaches have been proposed for the detection of various kinds of antibodies in human serum and other samples. Several polyclonal as well as monoclonal antibodies have also been proven to be practicable for the detection of organ-specific biomarkers of diseases in human serum and to detect human terminal complementary complexes in tissue and plasma samples [116,117,118]. Researchers have also discussed the possibilities of nucleic acid based aptamers for developing the ligands which have the potential for complementing the functionalities of long-established antibody-based technologies for future therapeutical and diagnostic advancements. The implication of the overexpression of IGF-1R in the growth, progress, metastasis, and therapeutical resistance of various cancers, viz., pancreatic, prostate, breast, and ovarian cancers, has been well documented in both pre-clinical and clinical reports. Drug candidates, e.g., monoclonal antibodies, showing direct interactions with IGF-1R include dalotuzumab, figitumumab, ganitumab, and cixutumumab, etc. Therefore, the non-invasive analyses of IGF-1R targeting antibodies in various cancer types would enable the classification of patients for personalized treatment options and could lead to improved outcomes both in the case of clinical studies as well as other routine treatment schedules [119].

There has always been a desperate requirement for ultra-sensitive detection techniques and efficient, as well as effective, therapeutical paradigms for both the treatment as well as the detection of stroke types in their infant stages. Carbon nanotubes have been one of the nanomaterials used to tackle some of these challenges. The loading capacity of the carbon nanotubes’ drug payload, viz., dexamethasone (DEX), and then their decoration with polyethylene glycol (PEG), as well as the antibody against the atrial natriuretic peptide (ANP), followed by the FITC (fluorescein iso-thiocyanate) to target the sites of an ischemic-stroke stroke-model of the rat, was studied by researchers. The CNTs functionalization with PEG, DEX, FITC, and ANP-antibody culminated in a 63-fold enhancement in D-band intensities, as seen by Raman spectroscopy. This specific enhancement in the intensity of the band was seen after the CNTs’ functionalization with DEX and PEG, as also established by the FTIR technique. These results also showed the coupling ability of the ANP-antibody and their detection capacity against DEX-PEG-CNTs [120]. This detection system was formulated, functionalized, and then finally characterized for its applicability in the therapeutic management and detection of ischemic-stroke.

Therapeutical drugs, as well as the monitoring of immunogenicity, are ever-increasing for guiding therapies with biologicals, and are characterized by higher individualized variabilities among the blood serum or plasma levels, for permitting non-subjective decisions in managing non-responding patients and for a reduction in the unnecessary intercessions with expensive therapeutic paradigms. However, therapeutical drug and immunogenicity monitoring has not yet been enrolled into clinic-based practices partly due to the uncertainty concerning the veracity, as well as the precision, of ELISAs (enzyme-linked immunosorbent assays). Researchers have studied the functionalization and delineation of some of the novel surface plasmon resonance (SPR)-based therapeutical drug and immunogenicity monitoring, which can be made applicable for measuring the serum concentrations of MAbs, viz., infliximab, which is an antibody against TNFα (tumor necrosis factor α), along with the anti-infliximab antibodies. Surface plasmon resonance possesses the apparent rewards of direct detection as well as the measurement of serum antibodies within the initial few minutes, and further avoids the longer incubation and/or separation, washing steps, and other longer detection processes from other methodologies which have been suggested so far, thereby aiding in the reduction in complexity and variability. Furthermore, drugs, as well as antidrug antibodies, could be concomitantly measured. These new methodologies were also further corroborated for their sensitivities and their reproducible character and established their cost-effectiveness as compared to the other commercialized ELISA kits. This methodology can also be made applicable to other biological therapeutic systems. These data can further increase the ways for developing SPR-dependent point-of-care tools and techniques for fast and accurate on-site detection systems [121].

The pharmacokinetic delineation of therapeutical antibody molecules has a significant role both in pre-clinical as well as clinical developmental stages. However, a precise pharmacokinetic assessment of these antibody molecules in serum from nonhuman primate models has often been frustrated by deficient specificities of assays for measuring the concentration of drugs. Researchers have described the application of a monoclonal antibody from a murine model used in the immunoassay arrangement for specific and quantitative measurement of human therapeutical antibody molecules in the non-human primates’ serum samples. This antibody molecule can be aimed against some distinct epitope located on the CH2-domain constant region among all isotypic human-IgGs. This antibody was specified as the antihuman Fc pan, and did not show cross-reactivity against various serum samples from cynomolgus monkeys, rats, mice, as well as non-human primates when employing the ELISA or surface plasmon resonance (SPR) technology (Biacore) format to measure various therapeutical antibody molecules. Applications of this antibody molecule, viz., antihuman Fc pan R10Z8E9, to capture and detect, can permit the quantification of human-specific entire therapeutical antibodies, viz., anti-IGF1R in serum samples of monkeys, through the sandwich ELISA technique. Moreover, a commercialized polyclonal antibody molecule aimed against Fc fragments of the human-Immunoglobulin-G can only distinctly quantify the therapeutical antibody molecules in the buffers, but not in the monkey serum samples. The present generic human-Immunoglobulin-G quantification test could already be employed in various pharmacokinetic assays carried out in monkeys for determining the concentration of therapeutic antibodies, which include anti-IGF1R as well. Substantiation of the assay for the human-Immunoglobulin-G therapeutical antibody molecule against the membrane proteins, demonstrated a decreased limit of measurements in the concentrated serum samples. The precision limits within, as well as among, the assays could be further characterized with a variation of less than 10% and the veracity was also reported to be within 15%. In conclusion, this monoclonal antibody molecule, viz., antihuman Fc pan R10Z8E9, can yield a standardized way to quantify the human-specific therapeutic antibody with higher sensitivities in the non-primates serum-samples, as well as in some generic human-Immunoglobulin-G assays [122].

For the interpretation of pharmacokinetics data of biological therapeutics, it has always been crucial to comprehend what drug molecules are being quantified by the pharmacokinetic assays. Concerning therapeutical antibody molecules, it has widely been admitted that freely disseminating antibody molecules have pharmacologically been active forms required for the determination of pharmacokinetics and/or pharmacodynamic kinships, assessments of the safety margin parameters, and for the projection of the doses from animal models to human studies and for the ultimate delineation of exposures in clinical settings. However, the entire drug payload can always be significant for the evaluation of dynamical interactions between a drug molecule and its various targets, as well as the entire exposure to the drug molecules. In the absence of, or with lower concentrations of, soluble ligands of the receptors, the total, as well as freely available, drug molecules, often become tantamount, and, hence, their estimation or detection becomes less sensitized to these various assays or the choices of the reagent to be employed for their detection. However, in the presence of appreciable quantities of the ligands, the designs of the assays and the delineation of reagents of these assays become crucial to comprehend the pharmacokinetics of the drug profiles. Additionally, various other reports have been published where diverse formats of assays affecting the quantified pharmacokinetic profiling, as well as data interpretation, have been performed. Several outcomes from the characterization of the reagents can furnish various possible explanations for ascertained discrepancies, highlighting the significance of characterization of the reagents for the comprehension of which therapeutic antibody molecules can be quantified for accurate interpretation of the pharmacokinetic profiles or parameters [123].

## 6. Sensing of Clinically Significant Macromolecules as Potential Biomarkers

**Figure 7:**
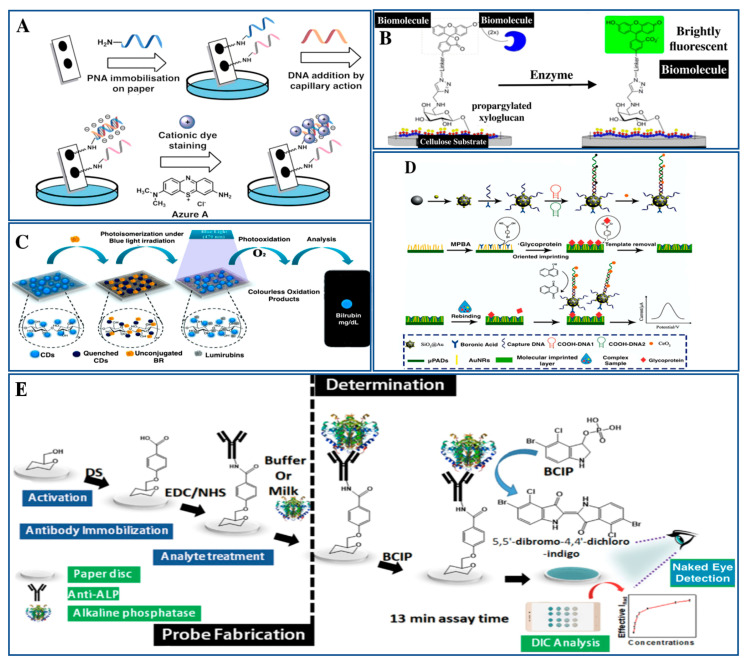
Paradigms and steps used in the sensing and detection of clinically significant bio-macromolecules for disease diagnosis. (**A**) DNA-based nanosensors fabrication by immobilizing acpc PNA (D-prolyl-2-aminocyclopentanecarboxylic acid—peptide nucleic acid) via covalent bond; (**B**) Surface tethering of the cellulose-based sensor for the identification of Esterase enzyme by using the flu orogen; (**C**) Illustration of bilirubin sensor using photoluminescent carbon dot sensing probes; (**D**) Fabrication pattern of the paper-based biosensor for determination of glycoprotein; (**E**) Schematic representation of a fabrication and detection method of ALP biosensor (adapted with permission from [124]).

Various advances have been made in the area of biosensing by modifying various matrices and their derived biomaterials. Recently, different approaches have been undertaken to formulate various nano-biomaterials for the fabrication of various kinds of biosensing platforms. Recent trends in using these nano-biomaterials in sensing and detection devices and biomolecular analytics techniques have shown appreciable enhancement due to their highly biocompatible and biodegradable nature, disposable properties, feasible portability, and cost-effective formulation. These nano-biomaterials also exhibit high potential for being developed into cell-based sensors due to their many distinct characteristics. Such nano-biomaterial-based sensors have been recently commercialized for their applications in many bio-medical diagnoses and have proven to be the first choice in clinical laboratories and personalized diagnostic procedures (Figure 7). The following are some of the approaches which have been recently employed for the sensing and detection of clinically significant bio-macromolecules for disease diagnosis.

### 6.1. Electrochemical Biosensing of Biomolecules

During the onset of disease, alterations in the concentrations of biological molecules often happen. There is a significant challenge in the measurement of such biomolecules, since these biological macromolecules are released quickly, in small quantities, from definite localities within complicated environmental conditions [125,126]. For meeting such complicated measurements, there has always been a need for sophisticated electrochemical assessment techniques and these have emerged as some of the significant approaches. Because of the different natures of the environmental conditions in which these assessments are carried out, a broad array of electrochemical sensing tools and techniques have been formulated for biomolecular quantification [127,128]. On that account, complicated customizations of the surfaces of electrodes have been performed for enhancing their electrochemical activities to detect these biological molecules. Furthermore, these electrochemical biosensing tools are chemical detectors where a biomolecule behaves as the sensing element. Several biomolecules have been employed for use in these biosensors, including but not limited to antibodies, carbohydrates, peptides and proteins, nucleic acids, etc. Enzymes are now being employed as the most highly used biomolecular detection element [129].

Two-dimensional materials have shown promising potential in their applicability in material sciences, in fields such as energy storing devices, environmental sciences, nanomedicines as well as biomedicines, sensing/biosensing techniques, and several other areas because of their particular physico-chemical and biological characteristics. Several researchers have shown various advancements achieved in the formulation of and fabrication of these 2D-material-based electrochemical sensing and biosensing platforms for their applicability in food safety and the detection of biomolecules, which are directly correlated with human health. For the purpose of biomolecular detection, bottom-up and top-down synthetic approaches of several two-dimensional materials, including graphene, oxides of transition metal elements, transition meta-dichalcogenide molecules, as well as various other materials have been introduced, and researchers have also established the structural and interfacial-chemistry of these two-dimensional materials, which has a critical role in their customization as well as the functionalization followed by their constitution with several other nano-scale building-blocks, viz., nanosized-particles, many polymeric systems, and other biomolecules. Furthermore, two-dimensional materials-based electrochemical sensing/biosensing to detect nitrites, ions of heavy metals, antibiotic molecules, and pesticidal agents in food and drinks has also been introduced into the market. Meanwhile, two-dimensional materials-based biosensors for the assessment and monitoring of significant other smaller molecules, which are linked to various aspects of human health and diseases, have also been introduced [130].

Electrochemical biosensors have been regarded as promising tools for the detection of biological molecules (viz., peptides and proteins, lipids, and nucleic acids, etc.), which are effective sources for the early diagnosis of various diseases. Advancements in electrochemical biosensing approaches have led to the evolution of many new types of biosensing platforms, thereby enabling the label-free, non-invasive detection of the viability, functions, and genetic patterns in whole cellular structures. Various reports have shown the enhancement of both the sensitivities as well as the specificities of electrochemical biosensors, which are among the most critical functionalities for the assessment of biosensor performances. Several nanomaterials, viz., metallic nanoparticles (NPs), carbon nanotubes (CNTs), graphene-based structures and their derivatives, and metal-oxide NPs, have been employed for improvement in the electrical conductivities and electro-catalytic capabilities of working electrodes, thereby enhancing the sensitivity of the biosensors. Several other customizations have also been carried out for advancing the specificities as well as the biocompatibility of biosensor platforms by employing biomaterials, viz., antibody molecules, aptamer-based molecules, and protein moieties from the extracellular matrix (ECM), and peptide-based composite structures. Many other recent electrochemical biosensors have also been designed for the detection of larger biological molecules and cancers as well as other stem cells from animal models [131].

Electrochemical biosensing platforms which have been paired to graphene-based quantum dot structures exhibit superior compatibility in strategies for cancer diagnosis, especially in the identification of alterations in the initial phase of tumorigenesis and for the detection of ultra-low levels of biomarkers that can differentiate between healthy non-cancerous and other cancer cells. Graphene-based quantum dots are classified among the novel classes of zero-dimensional semi-conducting nano-crystal systems and are diminutive graphene-based particles formatted in honey-comb-like structures with the nanoparticle size ranging from 1 to 50 nm. The particle size of these graphene-based quantum dots can be compared to the particle size of bio-macromolecules, hence, providing the ideal approach for studying biomolecules including viruses, peptides, proteins, etc. Graphene-based quantum dots have been high-ranking systems for specific as well as sensitive identification and detection of tumor markers, and they have been extremely interactive with other electrochemical biosensors [132].

In the last few decades, a good deal of research has been undertaken in the area of carbon-based nanomaterials on a global scale because of their notable thermal, chemical, mechanical, optical, and electronic characteristics. Various types of carbon-based nanomaterials, viz., including but not limited to carbon nano-horns, carbon-based nanofibers, carbon-based quantum dots, carbon-black, fullerenes, carbon nanotubes (CNTs), graphene-based nanostructures, nanodiamonds, etc., have shown constitutional characteristics which can be well utilized to develop and advance various technological approaches for biosensing platforms. Applications of nanomaterials within these biosensors have paved new ways and chances to detect analytes, as well as targeting biological macro- as well as micro-molecules. Carbon-based nanomaterials for electrochemical biosensors have been quite biocompatible, with improved sensitivities, better specificities, and lower detection limits for the detection of a wide array of chemicals as well as biological moieties. Several studies have been undertaken for the formulation and development of carbon-based nanomaterials for employment in electrochemical biosensors. The special characteristics of several of these nanomaterials, viz., carbon nano-horns, carbon-based nanofibers, carbon-based quantum dots, carbon-black, fullerenes, carbon nanotubes (CNTs), graphene-based nanostructures, nanodiamonds, etc., have also been improved alongside developmental advancements in their synthetic approaches as well [133].

Some of the earliest electrochemical 3D-printed biosensing platforms were formulated by the Belle group, where graphene-based biosensors were formulated over mylar-based substrates which was then followed by their being dipped into glucose-dehydrogenase liquids [134]. Some other types of enzyme-based biosensors used the immobilization of horseradish peroxidase (HRP) on the surfaces of graphene-based sensors. These sensors had the capacity for the selective detection of peroxides in human serum samples and showed stability for as long as 7 days [135]. DNA biosensors are another significant advancement in this area. To measure dopamine, pretreated graphene and PLA-based electrodes were employed, which were capable of monitoring the dopamine levels in human serum as well as human-serum-spiked samples [136].

Three-dimensional-printed biosensors have also been used for the observation of biological molecules in abrasive biological fluids. In the studies of Patel and coworkers, 3D molds compacted with carbon-based nano-composite electrodes were formulated and these were employed to detect the serotonin overflows from the whole of the colons of mice. Smaller metabolic biological molecules in biotic fluids, e.g., dopamine, glucose, and H_2_O_2_, with particular levels specifying definitive pathological anomalies, could function as biomarkers for early disease diagnosis [137].

Lately, Zhan and co-workers have formulated hybridized nano-spheres of LDHs and analyzed the electro-catalysis potential in oxygen reduction reactivities along with electrochemical sensors for their hydrogen peroxide (H_2_O_2_) detection capabilities. To fulfill the ongoing huge necessity of rapid point-of-care medical approaches, biosensors have been widely employed in angiogenesis, HIV-AIDS, and hereditary investigation, as well as in cancerous metastases, etc. Electrochemical biosensors are an extremely delicate platform to detect several biochemical as well as chemical target moieties and have been accordingly employed in various assays of cancer biomarkers’ detection. For several other kinds of different fatal disorders, H_2_O_2_ has been demonstrated to be the relevant molecular entity for accurately and promptly recognizing oxidative stress phenomena [138].

### 6.2. Optomechanics in Biomolecular Detection

Optomechanical sensing techniques have been developed based upon the coupling phenomenon between mechanized motion occurrence and optical-resonance, and have appealed to the vast interests in the area of sensor employability because of their small footprints, high sensitivities, low limits of detections, and electro-magnetic immunities. Optomechanical sensors, which have the capability of detecting mechanized motions at the nanometer scale to the vibrations of molecules at the same scale, have shown a wide array of applicability, viz., acoustic, chemical, and thermal sensing, inertia, force, etc. Several researchers have studied the various advancements in the formulation and characterization of optomechanical biosensors. Among all of these, three developmental areas of optomechanical sensors have been studied in greater detail: passive optomechanical biosensors, electro-optomechanical biosensing approaches, and molecular-level vibrating sensing systems. These biosensing configurations, their applicability, and integrative systems have also been researched in detail [139].

In the recent past, non-Hermitian systems have also been greatly researched in several areas, where the “exceptional point” (EP) became the indispensable character of these sensing systems which could be employed in the design of biosensors, for example, a mass sensing platform for the detection of a mass of nano-objects. This sensing system was prompted by the LIGO-based gravitational waves detection system by employing compacted or compressed light states. Researchers have tried enhancing the sensitivities of these mass sensors with nonlinear laser-based drives. These sensing systems are composed of two cavities in the optomechanical systems, which are mechanically conjugated or copulated and are nonlinearly driven by detuned laser systems (also called squeezed laser systems). In comparison to the case of linear drives, recent studies have yielded systems with higher sensitivities to mass. These sensitivity-increasing factors, along with the resonators’ optical damping, have been calculated and reported, and greater improvement has been observed consequently. These reports provide some wider perspectives on new quantum sensing systems, for their applications in the area of nanoparticulate detection systems, precision measurement, and improved sensing of other biological molecules [140].

Over recent decades, various platforms have been formulated to detect singular nanoparticles down to their molecular levels. Among these, optical detection systems found on higher-Q micro-cavities have shown appreciable utility for their high sensitivity and label-free operations. This optical-wave cyclization in micro-cavities is prepared to develop the radiation pressures which interrelate with the mechanized motions of these devices, and such optomechanical conjugation can prosper in complex physical environments. This has been widely explored in the recent past, in particular in the case of the quantum-control of microscopic mechanized motion [141]. Accordingly, in essence, these proposed cavities in optomechanical springs have been able to enhance the sensing resolution in comparison to conventional approaches. A sensitive approach to cavity optomechanics has been applied for mechanical displacement. To achieve single molecule resolution for nanomechanical sensing requires an extremely tiny mass of engaged nanomechanical oscillators. Particularly, stronger optical-spring effects from optomechanical conjugations give rise to some OMO frequencies, and these sensitively depend on the laser cavities’ de-tuning effects. These single molecular detections have now laid the foundations of some of the ultra-sensitive cavities in optomechanical spring sensing, and will lead to significantly improved sensing resolutions in the coming times [142].

Although the present focus has been on the particles and molecular-sensing approaches, the optical-based spring-sensing principles can be applied to other physical-sensing applicability such as electromagnetic-field-sensing platforms, gas sensing, and so on, which are dependent on detection systems of higher sensitivities in the context of the optical cavities’ resonance shifts, which are accelerated by extraneous physical disruptions. Therefore, researchers have demonstrated optical-spring sensing as having a broader applicability beyond the sensing of nanoparticles and biological molecules. One of the most prominent programs is the breakthrough in the detection of protein biomarkers when these proteins are shed from younger tumors into the bloodstream. The mechanics of the proteins, chiefly multiplex immunological assays, and mass spectroscopy have rapidly expanded in recent years with improved detection limits and multiplexing capabilities [143]. Researchers have also proposed biological detection systems that are dependent upon nanomechanical platforms for discovering, as well as detecting, cancerous proteins as biomarkers frequently seen in plasma samples. The operating modes of these detection devices have also been elucidated by researchers, and they have also put their focus on the latest advancements in the context of nanomechanical immunoassays (the sandwich systems) and nanomechanical spectrometric approaches. Technologies that can possibly have the capacity to detect tumor-related proteins, with their low abundance in the plasma in the very initial stages of cancers, have also been elucidated, among which, the first technique could allow the reproducible immunological protein detection in picogram concentrations, with a mean detection limit of 10 ag/mL. Biomarkers of tumors, including gene identification and proteomic alterations which are tumor-associated, have been crucial in the context of the preliminary choices of optimized anti-cancer therapeutics, disease detection, and more accurate and precise prediction of the progress of diseases [144].

Biomarkers for nucleic acids include mutations in genes, tumor-DNA in the circulation (ctDNA), genetic overexpressions, and chromosomal abnormalities. Accordingly, studies on the discovery of cancer biomarkers have been turning their attention to the discovery of proteinaceous biomarkers. In the initial stages, the proteinaceous biomarkers are released by tumors into the plasma and could prove to be the key to the initial detection of the tumor. In recent decades, nanotechnology-based approaches have provided a wide array of nano-biosensors that demonstrated ultra-high sensitivities and with smaller sample volumes. Several of these nano-biosensors exhibited various pitfalls and problems related to their specificities and reproducibility, as well as reliability, etc. The plasma proteome has presented the most all-encompassing sub-proteome for discovering biomarkers employed for the initial detection of tumors [145]. Label-free biosensing has exhibited crucial functionalities implicated in several health-related approaches. Micro and/or nano-photonic platforms have been widely researched for this intention and emerged as some of the potential approaches in recent times. Researchers have also proposed and demonstrated a technique that employs optical-spring effects in higher-Q tenacious optomechanical oscillators for an appreciable increase in the sensitivity resolution, by many orders of magnitude in comparison to traditional platforms, which could allow the detection of single bovine-serum albumin protein, having a molecular weight of 66 kDa, at a 16.8 signal-to-noise ratio. This particular optical-spring sensing platform has opened a distinct opportunity that can enable sensing of biomolecules and their recognition, along with a greater promise of broader physical-sensing applicability which relies on the extra-sensitive detecting capabilities of the optical-cavities’ resonance shifts for probing the extraneous physical characteristics [143].

### 6.3. Nanotechnology in Biomolecular Sensing

Nanotechnology has demonstrated an enhancing role in the formulation and development of biosensing platforms and the sensitivities and performances of these biosensors are upgraded by employing various nanomaterials in their formulations. The make-up of these nano-biomaterials has permitted the introduction of several new signaling as well as transduction techniques and approaches in biosensing platforms. By reason of their sub-micron-sized dimensional parameters, nanoprobes, nano-biosensors, and other nanotechnological systems have permitted rapid as well as simpler analysis in vivo. Nanotechnology refers to the study, creation, manipulation, and applications of nano-biomaterials, nano-systems, and nano-devices, generally with dimensional features smaller than 100 nm. In this way, in the development of biosensing platforms, nanotechnology has been playing an increasingly significant role. The use of many new signal transduction technologies in their manufacture allows the use of nanomaterials in biosensors, such as nano-sensors, nanoprobes, and submicron size and other nano-systems [129,146,147]. Nanoparticles have found a wide array of applicability in biosensors, viz., (a) functionalized nanoparticles, which include magnetic and electronic as well as optical NPs, are binding to biological macro- as well as micro-molecules (viz., nucleic acids, peptides, and proteins) and have advanced their application in biosensing platforms for the detection and amplification of many types of signals. Many of these nanoparticle-based biosensing platforms include acoustic as well as wave-based biosensors, optical, and magnetic biosensors, etc. (b) Acoustic wave biosensors have also been greatly employed to improve the limits of detection and sensitivity. (c) In bio-optical sensory devices, the increase in the resonance of metallic nano-clusters bonded to the surfaces by bio-cognitive actions has been demonstrated for the applications in biosensing platforms. (d) For the development of optical biosensors to detect and recognize specific DNA sequences, AuNPs (gold nanoparticles) were applied as a new class; they were also used for the quenching of the fluorescence. (e) Magnetic NPs are also versatile and powerful tools for diagnosis in biology and medicine, and these are often formulated either in the forms of singular domains or, due to their paramagnetic character, in the form of, e.g., maghemite, iron oxide, greigite, and several other kinds of ferrites, based upon their high transitional temperatures, and/or superconducting quantum interfering devices. (f) Colloidal AuNPs, due to their ultra-high surface areas, have been employed for enhancing DNA immobilization over gold-based electrodes, for the ultimate lowering of the limits of detection of formulated electrochemical DNA biosensors.

For the determination and detection of hydrogen peroxide, nanotechnological biosensors have shown higher sensitivities, long-term stabilities, and better reproducibility. For detecting hazardous chemicals in single cells, optical fibers with distal end diameters less than 10 microns, and with their surfaces coated with antibodies, were able to quantify the concentrations of benzopyrene-tetrol in human mammary cancer cells as well as in cells obtained from the epithelia of rats [148]. In the ongoing pandemic of COVID-19, more than 218 countries have been affected around 100 million people have been infected, with almost 2 million deaths reported by the beginning of 2021. Recently, vaccines were being formulated in China, European nations, the USA, Russia, etc., and some of these have been in the last stages of clinical trials, and on the waiting list for getting approved to be used by the general public. The other option which also seemed feasible was vigorous testing, isolating the infected individuals, and maintenance of the physical distance. Several methodologies have now been made available and are being formulated to test the suspected carriers and cases, so as to prevent the carriers of this virus. Efforts have also been undertaken for the development of nanotechnological approaches for both conventional and faster and more effective testing methodologies to diagnose COVID-19. These nanotechnological testing methodologies have also been based on target sensing, including viral RNA and spike-proteins, as well as antibodies, viz., IgG and IgM. Apart from the formulation of RNA-targeted polymerase chain reaction methods, antibodies, and pseudo-virus neutralizing assays, various other types of diagnosing technologies have also been formulated. Furthermore, nanotechnology-based biosensors are also being formulated for diagnosis of viral particles [149].

In the assays of α-fetoprotein, electro-chemiluminescence-based nanotechnological biosensing platforms are under formulation and development. There is another nano-structured biomaterial which has been researched considerably for nano-sensing uses, viz., nano-crystalline silicon, often known as porous-silicon, which has found various applications in optical-interferometric transduction to detect smaller organic molecular moieties, viz., digoxigenin and biotin, antibodies, and the protein streptavidin, at picomolar and/or femtomolar levels of the analytes [150,151]. In the context of cancer diagnosis and detection, earlier cancer detection has been quite critical for the initiation of anti-cancer therapeutics. The detection of cancers based upon the various types of biomarkers can efficiently enhance earlier predictions and the subsequent treatment strategies. Nanotechnology-based nano-biosensors to detect the biomarkers of cancers have been excellent platforms for molecular detection, as well as diagnosing this deadly disease. Several researchers have undertaken various advancements and accomplished the application of nanoparticles for detecting the biomarkers of cancer. Some of the recent advancements involve applying common nanomaterials, viz., gold, platinum, silver, iron oxide, carbon nanotubes, graphene, etc., along with newly emerging nanoparticles, viz., inorganic particles of zinc oxide, molybdenum, quantum dots, as well as MOFs (metal–organic frameworks) for diagnosing several of the biomarkers of colon, prostate, breast, and lung cancers [152].

The ingestion of microbially contaminated food may cause life-threatening health-related issues because of food-related disorders. Therefore, there has always been a necessity of more precise detection as well as identification of pathological micro-organisms and toxins present in food for the prevention of related concerns. A thorough comprehension of the concepts of biosensing has motivated researchers to formulate nano-biosensors by applying different nano-biomaterials and nano-composites for improvements in the sensitivities and specificities in their pathogen detecting approaches. The use of nano-biomaterials has also made researchers capable of applying advanced nano-technologies in biosensing platforms for transferring the signals for the enhancement of their efficiencies. Nanomaterials such as graphene-based nanomaterials, dendrimers, gold NPs, magnetic NPs, carbon nanotubes (CNTs), and quantum dots have been predominantly applied to develop biosensing techniques with better sensitivity and specificity of detection, mainly because of their exclusive physical, optical, magnetic, and chemical characteristics. All NPs and new nano-composites applied in biosensing technologies need to be categorized and classified to improve their performance, the speed of their detection capacities, and their unobtrusiveness, as well as their efficient application in food-borne disease detection and analysis [153].

Binding of ligands to receptors and DNA hybridization to micro-fabricate cantilevers leads to the production of surface-stress alterations which can be directly assessed to analyze various types of analytes [154]. Therefore, microcantilever-based DNA nano-biosensors have been formulated so as to be applied along with gold nanoparticle (Au-NP)-modified DNA. The application of nanoparticles has led to the designing and fine-tuning of their characteristics in several possible ways which cannot be possible with other kinds of nano-biomaterials. This can also allow the manipulation of their pharmacokinetic behavior and these nanoparticles need to have a diameter of more than 10 nm for avoiding single-pass renal clearance and should not possess a positive charge to any great extent (so as to minimize non-specific interactions with proteins as well as with cells). Nanoparticles should be large enough so as to possess several targeting ligands which could provide multi-valent binding sites to cellular surface receptors [155]. As these NPs gain entry into cells by the process of endocytosis, these can bypass the multi-drug resistance proteins and their mechanism, which involves the cellular surface protein-pumps, viz., glycoprotein-P (P-gP). For a better comprehension of the anatomy and functionality of biological systems at nanometer scales, nanotechnological approaches and other devices, including the confocal microscope, magnetic resonance imaging techniques, and two-phonon techniques are increasingly being applied [156].

## 7. Bioinformatics Tools and Statistical Criteria for Biomarker Analysis

Several bioinformatics tools are available for the analysis of biomarkers and stratification of cancer, including those that address the issue of cancer heterogeneity [157]. These tools include scRNA-Seq analysis tools, mutational signature analysis, tumor mutational burden analysis, network-based analysis tools, machine-learning-based tools, CytoTRACE, CIBERSORT, TumorMap, and MutSigCV [157,158,159,160]. Such tools are well known for analyzing and studying heterogeneity within the tumor and for understanding the variations in the mutations in the tumor genome [157]. In addition, the prediction of the progression of tumors based on predictive biomarkers has been enabled by implementing such bioinformatic tools. A brief introduction of some of the tools is discussed below.

**scRNA-Seq analysis tools**: Single-cell RNA sequencing (scRNA-Seq) can be employed for studying the heterogeneity of cancer cells within a tumor. It is a powerful and effective technique for studying gene expression patterns at the single-cellular level. It also enables scientists to understand genetic expressions and to identify the cell type and state. Notably, it provides explicit knowledge regarding novel gene expression patterns within the cell populations [161].

**Mutational signature analysis**: This tool is utilized to examine mutational signatures present in cancer genomes. These signatures refer to unique patterns of somatic mutations that develop due to particular mutational mechanisms [162]. There exist various tools that facilitate the analysis of mutational signatures, such as signature analyzer and mutational patterns [163].

**Tumor mutational burden analysis**: The tumor mutational burden (TMB) refers to the count of somatic mutations found in the genome of a tumor. In certain cancers, the TMB has been demonstrated to be an effective biomarker for determining the response to immunotherapy. A number of bioinformatics tools are accessible for evaluating the TMB, such as MSI sensors and TMB-hotspot tools [164].

**Network-based analysis tools:** Network-based analysis tools are useful in detecting cancer-related driver mutations and functional pathways [165]. These tools utilize Reactome pathway analysis to determine pathways that display significant accumulation of somatic mutations in a specific tumor. By doing so, Reactome aids in the identification of potential pathways and associated driver mutations involved in cancer development. This tool, therefore, is an effective way to discover key molecular pathways involved in tumor progression and identify novel therapeutic targets [166].

**Machine-learning-based tools:** There is a growing trend in utilizing machine-learning-based tools for the purpose of biomarker discovery and cancer stratifications [167]. Several machine-learning-based tools have been developed for cancer analysis, including DriverNet, Oncobox, and DeepCC. These tools leverage the power of machine learning algorithms to aid in identifying novel biomarkers that can serve as prognostic and predictive indicators of cancer development, progression, and response to therapy [168]. The integration of machine learning in cancer research is a promising approach that has the potential to improve patient outcomes by enabling the development of personalized and targeted treatment strategies [167].

**In silico biomarkers:** Recent staging systems for tumors are primarily based upon anatomical magnitudes of disorder. These require elaboration by biological characters for improving the classification of patients for anti-cancer therapeutics. Due to the advancements in genomics, transcriptomics, and bigger-data-based technologies, researchers can explore new molecular characteristic features of cancers in greater detail and assess their clinical significance. As a result, various prognostic and predictive genetic expressional signatures now possess potential for establishing the stratification of cancerous groups by biological determiners. These include several genetic expressions, RNA sequencing, interactome-aided algorithmic markers, in silico analysis of the molecular pathways of cancers, several in silico prognostic and survival biomarkers, etc. [169].

In silico investigations and assessments of the molecular pathways in cancer have recently been instrumental in helping to quantize many intracellular signaling activities, structural modifications, DNA damage, its synthesis and repairing, and several other cellular and molecular processes. This has deeper impacts in basic scientific research, biological industries, and medicines. Unlike the genetic ontological analyses and various other qualitative techniques which affirm whether the cancer signaling is affected in particular, the quantification approaches possess the benefits of assessing the extent of the cancerous pathway’s up and/or downregulation. This has resulted in the growth of new generations of activity assessments of molecular biomarker pathways and is reflected in terms of the concentration variations among all of these quantifiable pathway elements. These data are high-throughput proteomics-based or transcriptomics-based profiles, and the numerical outputs represent both the positive as well as negative parameters and reflect the generalized activation of cancerous pathways. In this context, researchers have described the concepts of in silico quantitative genetic expressional integration methodologies and their applicability in quantification of cancers’ molecular pathways. The researchers employ various bioinformatics tools as well as algorithms and their practical applicability to solve real world and practical problems. In addition to a profusion of their applicability in basic research, these in silico quantitative analyses of the cancers’ molecular pathways lead to improvements in the molecular patterns and clinical assessments in industries, and can aid in the finding of new active biotechnological and pharmaceutical compounds and hence appreciably help in the progress and evolution of customized therapeutic regimens. In addition to these principles of theory and conceptualized paradigms, researchers also employ various open and available software, applicable for large-scale data of the expressions of proteins and/or RNAs, for assessing the levels of upregulation and downregulation of several cancer pathways [170].

In the case of neuronal cancers, genetic expression of specific biomarker genes has a strong correlation with the survival of patients and is often found to exceed the histological evaluations. Employing human models of the interactome, researchers have algorithmically reconstructed several new molecular cascades that are focused on individualized proteins. Each of these singular genetic expressions and activations of the gene-regulated pathways could be evaluated as a criterion for a patient’s survival and for grading of the biomarkers of tumors in neuronal cancers including diagnosis-based subgroups. Researchers have used some of these data from The Cancer Genome Atlas, which includes several incidences of high-grade neuronal cancers and some cases of low-grade neuronal cancer profiles. They then distinguished these specific genes along with their molecular pathways as the biomarkers for the patients’ survival. Researchers have also evaluated the various grades of neuronal cancers along with the molecular biomarkers of the cancer-subtypes having a threshold of AUC > 0.7. The outcomes of these studies have suggested an efficacy of these approaches of the reconstructed pathways that is nearly twice that of the genetic biomarkers [171].

**Statistical criteria for validation of biomarkers:** Prognostic biomarkers can be distinguished through chief effective tests of the correlation between these biomarkers and their outcomes in several statistical models. An example of such a prognostic biomarker is a mutation in some gene that has been correlated with insufficient outcomes in non-squamous small cell lung cancer. Likewise, predictive biomarkers can be distinguished in sub-altern analyses by employing the data from several randomized and controlled clinical studies, through the interaction tests carried out between the therapeutic regimens and respective biomarkers in various statistical models. Table 2 provides several other criteria for the application of metrics for assessing the performance of biomarkers.

## 8. Radiomic Markers

Radiomic markers are a type of biomarker that uses quantitative analysis of medical images to extract features related to tumor morphology, texture, and spatial distribution [173]. These features can be used to develop mathematical models predicting clinical outcomes, such as tumor response to treatment, disease progression, or patient survival [174]. Radiomic markers possess many potential roles as biomedical bioindicators, including early detection and diagnosis of disease, patient stratification, treatment monitoring, and prognostic prediction [175]. In early detection and diagnosis, the subtle changes in tumor tissue are also readily diagnosed from the morphology of tissue (size and shape). These markers are an effective strategy and are successful in detecting abnormal growth which could be identified by the naked eye. In addition, in reference to patient stratification, these markers are helpful in grouping patients based on tumor phenotype of patients, which enables more personalized chemotherapy [176]. Even the response to chemotherapy and to what extent a patient is at risk for reoccurrence of cancer could be identified by these markers. Notably, the early signs of resistance development to the treatment response could also be identified by these radiomic markers. In addition, these markers predict the patient outcomes based on survival, or disease-free survival, based on the tumor phenotype [177].

Regarding the associations between molecular biomarkers and radiomic markers, there is growing evidence that these two types of biomarkers are closely related [178]. Molecular biomarkers can provide information about the underlying biological mechanisms that drive tumor growth and progression, while radiomic markers can provide information about the resulting changes in tumor phenotype. By combining molecular and radiomic biomarkers, a more comprehensive understanding of tumor biology and accurate prognostic and predictive models could be developed [179].

## 9. Conclusions and Future Perspectives

A biomarker is employed for understanding cardinal and key biological processes and their correlation with disease pathophysiologies and therapeutic regimens. A particular biomarker for diagnosis and prognosis of a disease and the responses of the disease to various therapeutic options is applied in the screening of plasma, serum, and tissue samples, and function as new tools for developing therapeutic regimens. Employing a specific biomarker for screening the patients or subjects for determining the eligibility of the clinical studies, and in the initial toxicology trials, holds the capacity for decreasing the failure rates of drugs in the later phases of clinical trials. Conventional research methodologies are being used for studying the biomolecules, proteins, peptides and genes, as well as other metabolites of concerns. Additionally, various existing and new tools are also being formulated especially for being employed in the investigation in pre-clinical as well as clinical stages of drug discovery and development. More significantly, transitioning the pharmacological compounds and drugs from pre-clinical phases to the of clinic is further assisted by techniques which are capable of bridging both the procedural segments. Identifying biomarkers which can be researched during the entire developmental process necessitates tools and techniques which are executable and economical for larger patient sample sizes.

In brief, biomarkers have always been an integral component of comparatively new and idealized clinical tools for prognosis, treatment, as well as diagnosis of several diseases. There have been important advantages of employing biomarkers for studying several aspects of diseases and their therapeutic regimen, drug discovery and development, and to monitor possible efficacies of treatment interventions. As compared to recent measurements, a biomarker is required to furnish the tests with higher sensitivities and specificities, help to improve decision-making procedures, and aiding in facilitating the formulation and development of therapeutic interventions. For improving healthcare and producing cutting-edge therapeutical paradigms, several efforts are being undertaken for exploring the biomarker-based frontiers for searching for new and/or improved biomarkers. However, because the process which leads to pathogenesis of a disease is frequently quite complicated, it has become comparatively feasible to describe, discover, and distinguish useful biomarkers to assess the responses of drugs, frame the disease diagnoses, and track the disease development, which can better detect underlying anomalies affiliated with disease as well as the pharmacological mechanism of action of several drugs. For basic, as well as clinic-based pharmacologists, and other professionals participating in biomarker identification, collection of these data and information has always been a challenge. Identifying the differences between possible biomarkers and other authentic biomarkers which could be universally employed for guiding the crucial clinic-based choices has been among the chief challenges in the biomarker arena. Efficient biomarkers must determine clinic-based evaluations for improving patients’ care. Clinical conclusions which are dependent upon the real test outcomes should possess more advantages compared to others which are based upon false-negative or false-positive outcomes. A biomarker must also be economic in terms of the reduction in the associated cost and adverse effects, while also considering death prevention and risk management. The efficacies and efficiencies of biomarkers are ascertained by their comparison with ideal biomarkers and delving into their properties.

A promising biomarker must have characteristic features identical to the properties of an ideal biomarker. Although a large number of new biomarkers are being employed, only a handful of these have proven to be very efficient in clinical settings. The challenges and troubles faced while discovering and validating a biomarker are many and can be disvalued when undertaking the development of schemes and carrying out experimental analyses. For obtaining assuring outcomes, researchers should employ annotated clinical samples, use the appropriate groups as the control in experiments, use larger sample sizes, and standardize the specimen handling techniques as well. In coming times, the integration of the biomarkers currently distinguished and described, with the evolving high-throughput technologies in practices of disease and medicine, will be necessitated for achieving personalized treatment options and prevention of diseases.

Future efforts should focus on exploring some of the non-invasive, highly specific, and sensitive biomarkers having high diagnostic value, simplifying the evaluation and assessment of the candidature of biomarkers for their introduction into clinical practices, undertaking the formulation and development of new algorithm-based approaches as well as bioinformatics tools and software to explore the molecular-level interacting phenomena correlated with metabolic modifications, and demonstration of the effective and efficient affiliations in terms of the integration of the laboratory as well as clinical outcomes. In addition, more intensive and further delineation of smaller molecules and body metabolites correlated with the gut microbiota could reveal many more metabolic characters linked to phenotypical variabilities in the pathogenesis of diseases, enabling assistance in the selection of strategies for disease prognosis, diagnosis, and disease treatment, and realization of the profits and gains which these smaller molecular metabolites can impart to the precision and to personalized therapeutic regimens. Smaller molecular metabolite-based metabolomic strategies have appreciably altered the scene of biomedical exploration. In the near future, metabolite-based biomarker molecules will undergo effective validation and then their transfer to clinical practice can be undertaken, therefore, researchers are required to work in close correlation with clinicians. To enhance the accessibility of metabolite-based biomarker molecules, their analytical instruments are required to be economic and simplified, providing deeper insights into the particular metabolic patterns.

## Figures and Tables

**Table 1:** Classification of new omics-based biomarkers (adapted from [14]).

Biomarkers	Classification
Epigenetic	Biomarkers based on epigenetics, e.g., DNA methylation, histone modification, non-coding RNAs
Genetic	Biomarkers based on changes in DNA, e.g., polymorphism of a single nucleotide (SNP)
Lipidomic	Biomarkers based on the lipid profile
Metabolomic	Biomarkers based on the metabolic profile
Proteomic	Biomarkers based on the protein profile
Transcriptomic	Biomarkers based on RNA profile, e.g., expression of RNA

**Table 2:** Various metrics applied for the assessment of the performance of biomarkers (adapted from [172]).

Metrics	Details
Sensitivity	The proportion of cases that test positive
Specificity	The proportion of controls that test negative
Positive predictive value	Proportion of test-positive patients who actually have the disease; is a function of disease prevalence
Negative predictive value	Proportion of test-negative patients who truly do not have the disease; is a function of disease prevalence
ROC (plot of sensitivity (true-positive rate) versus 1–specificity (false-positive rate), with a data point calculated for every value of the marker in the data set) curve	Plot of sensitivity (true-positive rate) versus 1–specificity (false-positive rate), with a data point calculated for every value of the marker in the data set
Discrimination	How well the marker distinguishes cases from controls; often measured by the area under the ROC curve; ranges from 0 to 1, with 0.5 indicating performance equivalent to a coin flip and 1 corresponding to perfect ability to distinguish
Calibration	How well a marker estimates the risk of disease or of the event of interest

## Data Availability

Not applicable.
